# Afipia dichlorophenoxyacetatis sp. nov., isolated from field soil in Japan, degrades 2,4-dichlorophenoxyacetic acid

**DOI:** 10.1099/ijsem.0.006672

**Published:** 2025-02-10

**Authors:** Hiroyuki Sawada, Yoriko Sakai, Yusuke Takashima, Ken Naito, Mitsuo Horita, Mamoru Satou

**Affiliations:** 1 Research Center of Genetic Resources, National Agriculture and Food Research Organization (NARO), 2-1-2 Kannondai, Tsukuba, Ibaraki 305-8602, Japan; 2 Institute for Agro-Environmental Sciences, NARO, 3-1-3 Kannondai, Tsukuba, Ibaraki 305-8604, Japan

**Keywords:** 2,4-D, *cad*, microbial degradation, phylogenomic analysis, polyphasic taxonomy, *tfd*

## Abstract

Bacterial strains, designated DD3^T^ and DDX28, were isolated from field soil in Japan. The strains had the ability to use 2,4-dichlorophenoxyacetic acid as the sole carbon source. They were Gram-reaction-negative, oxidase-positive, weakly catalase-positive, aerobic and non-spore-forming. Their cells were rod-shaped and often lacked flagella, but some exhibited motility due to the presence of one or two polar flagella. The genomic DNA G+C content was 58.8 mol%, and the major cellular fatty acids (>10% of the total fatty acids) were summed feature 8 (C_18 : 1_* ω7c* and/or C_18 : 1_* ω6c*), C_18 : 0_ and C_17 : 0_ cyclo. Phylogenetic analyses based on *gyrB* gene sequences and phylogenomic analysis using whole-genome sequences confirmed that the strains belong to the genus *Afipia*; however, their phylogenetic position did not match that of any known species of this genus. Comparative studies of the average nucleotide identity and digital DNA–DNA hybridization with closely related species revealed values lower than the thresholds used for prokaryotic species delineation (95–96 and 70%, respectively), with the highest values observed for *Afipia broomeae* ATCC 49717^T^ (79.92 and 21.5%, respectively). Phenotypic characteristics, cellular fatty acid composition and specific metabolic processes and biosynthetic gene clusters could differentiate the strains from their closest relatives. Our phenotypic, chemotaxonomic and genotypic data indicate that DD3^T^/DDX28 constitute a novel *Afipia* species, for which we propose the name *Afipia dichlorophenoxyacetatis* sp. nov., with DD3^T^ (MAFF 311804^T^=ICMP 25015^T^) as the type strain.

## Introduction

The genus *Afipia*, first proposed in 1991 by Brenner *et al*. [1] and validated in 1992 [2], belongs to the family *Nitrobacteraceae* of the order *Hyphomicrobiales*. To date, six validly named species were accommodated within the genus according to the List of Prokaryotic Names with Standing in Nomenclature (https://lpsn.dsmz.de) [3]. *Afipia felis*, *Afipia broomeae* and *Afipia clevelandensis* have been isolated from human clinical specimens and are considered potentially relevant to human diseases [1,4]. However, *Afipia birgiae* and *Afipia massiliensis* isolated from hospital water supplies and *Afipia carboxidovorans* isolated from wastewater are not associated with any human or animal diseases [5,7].

A chlorinated aromatic compound, 2,4-dichlorophenoxyacetic acid (2,4-D), has been used worldwide as a herbicide to control broadleaf weeds since the 1940s. It is readily degraded by micro-organisms, and the underlying mechanisms of its microbial degradation have been extensively studied [8]. Notably, 2,4-D degradation genes are often located in mobile genetic elements, making 2,4-D degraders model micro-organisms for horizontal gene transfer studies [9,11].

To study the diversity of 2,4-D degradation genes and related mobile genetic elements, several strains that can grow on media containing 2,4-D as the sole carbon source have been isolated from agricultural soil in Japan [10] and deposited in the Genebank Project (https://www.gene.affrc.go.jp/databases-micro_search_en.php) of the National Agriculture and Food Research Organization, Japan. Specifically, as the first *Afipia* strain utilizing 2,4-D as the sole carbon source, strain DD3^T^ was isolated from non-paddy agricultural soil in Ibaraki Prefecture, Japan, in 2005 [12]. Subsequently, another 2,4-D-utilizing *Afipia* strain, DDX28, was obtained in the same manner from the same soil sample, which had been stored at 4 °C for ~1 year and then pre-incubated with 2,4-D at 28 °C for 2 weeks. Genome sequencing of DD3^T^ as a representative 2,4-D-utilizing *Afipia* strain confirmed the presence of 2,4-D degradation genes in its chromosome [12]. Furthermore, the *cad* gene cluster, involved in the upstream pathway through which 2,4-D is converted to chlorocatechol, and the *tfd* gene cluster, involved in the modified *ortho*-cleavage pathway through which chlorocatechol is completely degraded, were found to accumulate as a single gene cluster of ~15 kb, constituting the 2,4-D degradation genes in DD3^T^ [12].

In this study, we aimed to clarify the taxonomic affiliations of DD3^T^ (MAFF 311804^T^=ICMP 25015^T^) and DDX28 (MAFF 311814=ICMP 25016). We conducted a detailed comparative analysis of these strains and their related species using a polyphasic approach and found that they constitute a novel species of the genus *Afipia*, which we termed as *Afipia dichlorophenoxyacetatis* sp. nov.

## Genome features

The complete genome sequence of DD3^T^ has already been determined (Table S1, available in the online Supplementary Material), and its characterization was reported in the previous study [12]. In this study, the DDX28 genome was sequenced using nanopore technology. The strain was cultured on buffered charcoal-yeast extract (BCYE) agar (Becton, Dickinson and Company) at 30 °C for 14 days, and the collected bacterial cells were used for DNA extraction. High-molecular-weight genomic DNA was extracted and purified using the NucleoBond HMW DNA kit (Macherey-Nagel), according to the manufacturer’s instructions. Purified DNA (400 ng) was used for library preparation with the Rapid Barcoding Kit SQK-RBK004 (Oxford Nanopore Technologies). Long-read sequencing was performed using the PromethION platform with R9.4.1 PromethION flow cell FLO-PRO002 (Oxford Nanopore Technologies). Using MinKNOW version 23.04.5 and Guppy version 6.5.7 (Oxford Nanopore Technologies), nanopore sequencing signals were subjected to real-time basecalling with a high-accuracy model, demultiplexing, adapter trimming and low-quality filtering with the following thresholds: >1000 bases and >7 Phred quality scores. The obtained raw reads (33 519 reads; 199 Mb; *N*_50_=9.6 kb) were *de novo* assembled using Flye version 2.9.2 [13] with default parameters. The resulting assembly was further polished using Medaka version 1.8.0 (parameter: ‘-m r941_prom_hac_g507’) (https://github.com/nanoporetech/medaka) with the filtered reads (25 494 reads; 147 Mb; *N*_50_=9.7 kb) obtained by filtering the raw reads with >10 Phred quality score cut-off and trimming 100 bases from both ends of the respective reads using chopper version 0.5.0 [14]. Finally, a 5 959 441 bp circular chromosome with 33-fold coverage and 58.8 mol% DNA G+C content was obtained, which was subsequently annotated using the DDBJ Fast Annotation and Submission Tool (DFAST) pipeline version 1.2.20 (https://dfast.ddbj.nig.ac.jp) [15], yielding two rRNA operons, 51 tRNA genes and 5876 protein-coding sequences (Table S1).

To taxonomically evaluate the relationship between DD3^T^ and DDX28, digital DNA–DNA hybridization (dDDH) analysis was performed using their genome sequences with formula 2 of the Genome-to-Genome Distance Calculator 3.0 (GGDC3) (https://ggdc.dsmz.de/ggdc.php#) [16,17]. A high similarity of 99.9% was observed for the strains, which is above the threshold (70%) for the delineation of prokaryotic species [18].

To preliminarily assess the taxonomic identity of DD3^T^/DDX28 based on genome analyses, the Type Strain Genome Server (TYGS) (https://tygs.dsmz.de) [19] and Taxonomy Check implemented in DFAST [15] were used. The genome sequence data of DD3^T^ were uploaded to TYGS to calculate the dDDH index and identify its closest type strain. The dDDH values calculated against *A. broomeae* ATCC 49717^T^ and *A. massiliensis* DSM 17498^T^ were the highest (21.5%; Table S2), but they are below the threshold value for prokaryotic species delineation [18]. According to Taxonomy Check using the fast average nucleotide identity algorithm [20] (Table S3), the closest relatives to DD3^T^ were *A. broomeae* ATCC 49717^T^ [80.036% average nucleotide identity (ANI)] and * A. massiliensis* DSM 17498^T^ (80.034% ANI), but their ANI values are lower than the cut-off value (95–96%) for prokaryotic species delineation [18]. These results suggest that DD3^T^/DDX28 may constitute a novel species of the genus *Afipia*.

## 16S rRNA gene analyses

To comprehensively identify the bacterial species closely related to DD3^T^/DDX28, a homology search based on 16S rRNA gene sequences was conducted as previously described [21,22]. Briefly, partial sequences (1420 bp) of the strains were determined via direct sequencing of their PCR products, which showed that the two sequences matched. Furthermore, these sequences were consistent with those of the 16S rRNA genes present in two copies in the genomes of DD3^T^ and DDX28. A homology search was conducted using the EzBioCloud database (https://www.ezbiocloud.net/identify) [23] with the DD3^T^ sequence used as a query. A total of 38 known species showing high homology with DD3^T^ were identified, and their 16S rRNA gene sequences were collected from the EzBioCloud database. Next, the 16S rRNA gene sequences of the species exhibiting high dDDH/ANI values with DD3^T^ in the aforementioned genome analyses performed using TYGS and DFAST (Tables S2 and S3) were retrieved from GenBank. Then, using a pairwise nucleotide sequence alignment tool (https://www.ezbiocloud.net/tools/pairAlign) [23] in EzBioCloud, their similarity values against the 16S rRNA gene sequence of DD3^T^ were calculated. Finally, by combining the data derived from EzBioCloud, TYGS and DFAST and eliminating duplicates, 46 known species with validly published and correct names were determined to be closely related to DD3^T^/DDX28 (Table S4). Notably, *Bradyrhizobium jicamae* PAC68^T^ showed the highest sequence similarity (98.23%) with DD3^T^.

To roughly know the phylogenetic position of DD3^T^/DDX28, phylogenetic analyses based on 16S rRNA gene sequences were carried out as described in our previous studies [21,22], targeting DD3^T^/DDX28, the 46 species (closely related to DD3^T^/DDX28; Table S4) and *Pseudorhodoplanes sinuspersici* RIPI 110^T^ (selected as an outgroup based on the analysis result of Hördt *et al*. [7]). The 16S rRNA gene sequences were analysed using mega 11 version 11.0.13 [24] with the neighbour-joining, maximum-likelihood and maximum-parsimony methods. The reliability of the tree was tested using the standard bootstrap method with 1000 replications. In the resulting phylogenetic trees, DD3^T^ and DDX28 clustered as a monophyletic clade with a bootstrap value of 100% (Fig. S1). However, the phylogenetic position of this clade did not match that of the known species used for analysis.

## *gyrB* gene analyses

The phylogenetic position of DD3^T^/DDX28 was evaluated using *gyrB* gene sequences. Of the 46 species identified to be closely related to DD3^T^/DDX28 (Table S4), 41 species (Table 1), excluding five species (*Bradyrhizobium erythrophlei*, *Bradyrhizobium namibiense*, *Bradyrhizobium ripae*, *Bradyrhizobium americanum* and *Bradyrhizobium subterraneum*) whose genome sequences were not publicly available, were used for comparison with DD3^T^/DDX28. The *gyrB* gene sequences of DD3^T^/DDX28, *Pseudorhodoplanes sinuspersici* RIPI 110^T^ (outgroup) and the 41 closely related species (Table 1) were extracted from their respective genome sequences, and phylogenetic analyses were conducted as previously described [25].

**Table 1.** Genomic relationship between *A. dichlorophenoxyacetatis* sp. nov. strain DD3^T^ and the type strains of the closely related species The ANI algorithm using blast (ANIb), orthologous ANI algorithm using USEARCH (OrthoANIu) and dDDH values were calculated using the genome-based distance matrix calculator [27], ANI calculator [28] and GGDC3 (formula 2) [16,17], respectively. Here, the data are presented in a descending order of their ANIb values calculated against the DD3^T^ genome sequence.

Species	Strain	Accession number*	ANIb (%)	OrthoANIu (%)	dDDH (%)
***A*. *dichlorophenoxyacetatis***	DD3^T^	AP029250.1	100.00	100.00	100.0
***A. dichlorophenoxyacetatis***	DDX28	AP029255.1	99.98	99.96	99.9
*A. broomeae*†	ATCC 49717^T^	AGWX00000000.1	79.92	77.94	21.5
*A. birgiae*†	34632^T^	CAJQ00000000.2	79.88	77.93	21.5
*A. massiliensis*†	DSM 17498^T^	NZ_JACHIJ000000000.1	79.77	78.06	21.5
*A. clevelandensis*†	ATCC 49720^T^	AGWY00000000.1	79.64	77.77	21.2
*A. felis*†	NCTC 12499^T^	UFSI00000000.1	79.35	75.07	20.6
*A. carboxidovorans*†	ATCC 49405^T^	NC_011386.1	79.21	75.15	20.8
*Pseudomonas carboxydohydrogena*	DSM 1083^T^	NZ_CP113162.1	79.16	75.37	20.7
*Bradyrhizobium algeriense*	RST89^T^	PYCM00000000.1	78.86	74.86	21.5
*Bradyrhizobium jicamae*	PAC68^T^	NZ_LLXZ00000000.1	78.75	75.20	21.1
*Bradyrhizobium sediminis*	S2-20-1^T^	NZ_CP076134.1	78.68	75.53	20.6
*Bradyrhizobium elkanii*	USDA 76^T^	ARAG00000000.1	78.66	75.23	20.8
*Bradyrhizobium pachyrhizi*	PAC 48^T^	LFIQ00000000.1	78.65	75.09	21.1
*Bradyrhizobium lablabi*	CCBAU 23086^T^	LLYB00000000.1	78.62	75.15	20.8
*Bradyrhizobium mercantei*	SEMIA 6399^T^	MKFI00000000.1	78.62	75.00	20.9
*Bradyrhizobium ivorense*	CI-1B^T^	CAADFC000000000.2	78.56	75.25	20.9
*Bradyrhizobium viridifuturi*	SEMIA 690^T^	LGTB00000000.1	78.56	75.10	20.7
*Bradyrhizobium paxllaeri*	LMTR 21^T^	NZ_CP042968.1	78.53	75.14	20.9
*Bradyrhizobium tropiciagri*	SEMIA 6148^T^	LFLZ00000000.1	78.51	75.20	20.7
*Bradyrhizobium japonicum*	USDA 6^T^	NC_017249.1	78.51	74.62	20.6
*Bradyrhizobium embrapense*	SEMIA 6208^T^	LFIP00000000.2	78.50	75.09	20.8
*Bradyrhizobium icense*	LMTR 13^T^	NZ_CP016428.1	78.50	74.88	20.8
*Bradyrhizobium liaoningense*	NBRC 100396^T^	BSOX00000000.1	78.49	74.66	20.2
*Bradyrhizobium cajani*	1010^T^	NZ_WQNE00000000.1	78.49	74.77	20.8
*Bradyrhizobium septentrionale*	1S1^T^	NZ_CP088285.1	78.45	75.01	20.8
*Bradyrhizobium neotropicale*	BR 10247^T^	LSEF00000000.1	78.45	74.79	20.6
*Bradyrhizobium murdochi*	WSM 1741^T^	NZ_AXAU00000000.1	78.45	74.86	20.6
*Bradyrhizobium retamae*	Ro19^T^	LLYA00000000.1	78.45	75.02	20.6
*Bradyrhizobium archetypum*	WSM 1744^T^	NZ_JAAVLW000000000.1	78.43	74.95	20.6
*Bradyrhizobium shewense*	ERR11^T^	FMAI00000000.1	78.43	74.66	20.6
*Bradyrhizobium stylosanthis*	BR 446^T^	LVEM00000000.1	78.39	74.81	20.5
*Rhodopseudomonas rhenobacensis*	DSM 12706^T^	NZ_JACHIH000000000.1	78.39	75.38	20.7
*Bradyrhizobium ottawaense*	OO99^T^	NZ_CP029425.1	78.36	74.59	20.4
*Bradyrhizobium canariense*	BTA-1^T^	NZ_VSST00000000.1	78.36	74.44	20.4
*Bradyrhizobium rifense*	CTAW71^T^	VSSS00000000.1	78.32	74.85	20.4
*Bradyrhizobium diazoefficiens*	USDA 110^T^	NC_004463.1	78.31	74.80	20.6
*Bradyrhizobium huanghuaihaiense*	CGMCC 1.10948^T^	VLLA00000000.1	78.31	74.82	20.6
*Bradyrhizobium betae*	PL7HG1^T^	NZ_CP044543.1	78.28	74.97	20.1
*Bradyrhizobium australiense*	WSM 1791^T^	NZ_JAAVLX000000000.1	78.25	74.74	20.6
*Bradyrhizobium daqingense*	CGMCC 1.10947^T^	VLKL00000000.1	78.23	74.74	20.2
*Bradyrhizobium diversitatis*	CNPSo 4019^T^	NZ_JACEGD000000000.1	78.23	74.72	20.5
*Bradyrhizobium cenepequi*	CNPSo 4026^T^	NZ_JAGKJI000000000.1	78.20	74.85	20.3

**Fig. 1.**
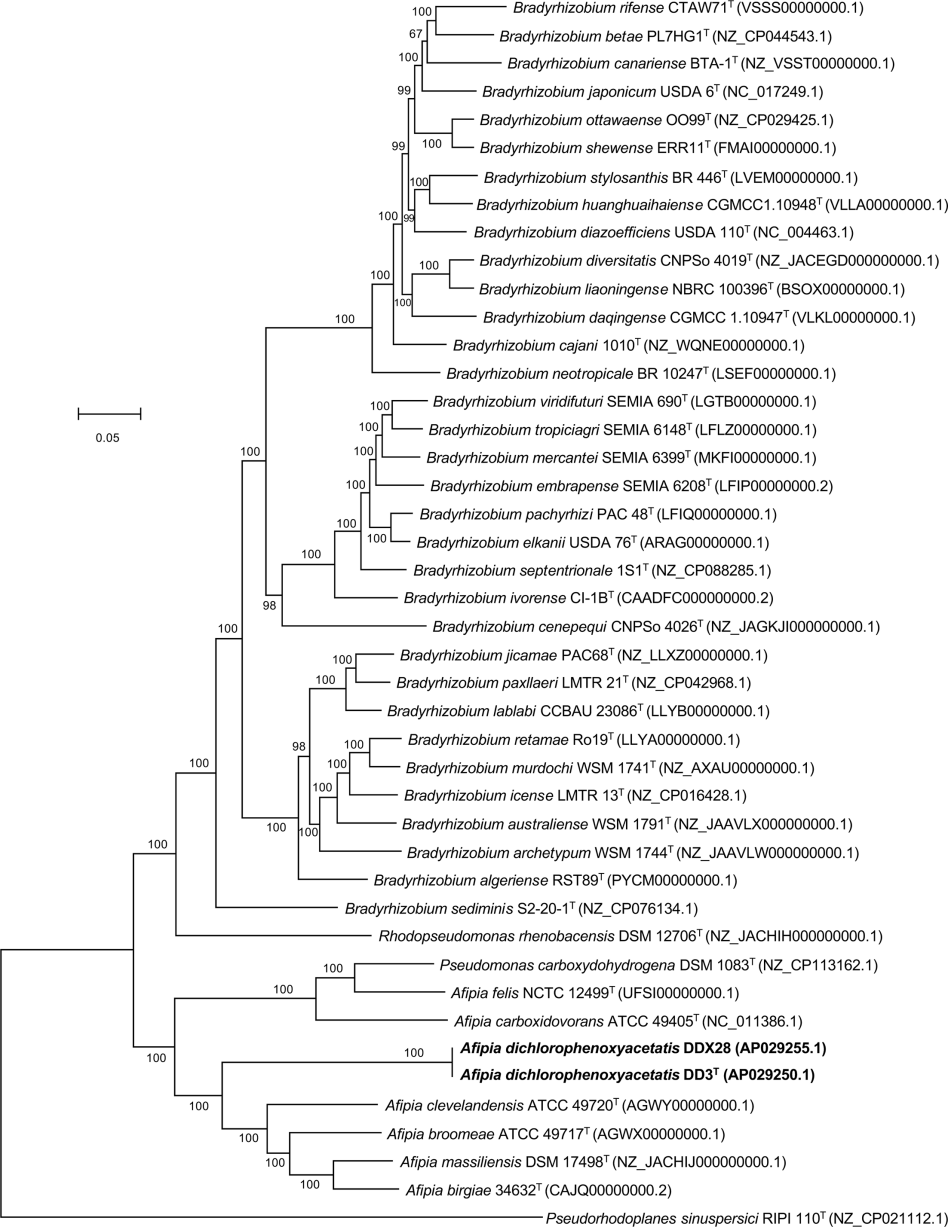
Phylogenomic tree reconstructed based on the concatenated alignment of 475 core genes (total alignment length was 334 305 bp), showing the relationships between *A. dichlorophenoxyacetatis* sp. nov. strains (boldface type) and the closely related species listed in Table 1. The concatenated alignment was generated using Roary [30] and Gblocks [32]. The maximum-likelihood tree was inferred using RAxML-NG [33] with a general time-reversible substitution model and a gamma model of rate heterogeneity. *Pseudorhodoplanes sinuspersici* RIPI 110^T^ served as an outgroup. GenBank accession numbers are shown in parentheses. T, type strain of the species. Numbers at nodes indicate the standard bootstrap values from 100 replications.

* The whole-genome sequences used here are the same as those used in the phylogenomic analysis (Fig. 1).

† The type strains of these six species were selected for comparison with DD3T/DDX28 in the phenotypic and chemotaxonomic studies (Tables2  3) and functional genomics (Figs 3 and S4, Table S6).

DD3^T^ and DDX28 clustered tightly (Fig. S2), similar to the results of the 16S rRNA gene sequence analyses (Fig. S1). Furthermore, these two were clustered together with *Pseudomonas carboxydohydrogena* and six known species of the genus *Afipia* as a monophyletic clade, with a standard bootstrap value of 100%. However, the phylogenetic position of DD3^T^/DDX28 was not consistent with any other member of the clade. Note that Anzai *et al*. [26] reported that *Pseudomonas carboxydohydrogena* is not a member of the genus *Pseudomonas* but part of the ‘*Bradyrhizobium* group rRNA lineage’.

## Comparative genomics and phylogenomics

Whole-genome similarity was analysed by calculating the ANI and dDDH values between DD3^T^ and its closely related species. Briefly, the ANI values were determined with the ANIb (http://enve-omics.ce.gatech.edu/g-matrix/index) [27] and OrthoANIu (https://www.ezbiocloud.net/tools/ani) [28]. The dDDH values were computed using formula 2 of GGDC3 [16,17]. The genome sequence of DD3^T^ was used as a query, and the 41 closely related species (Table 1) with publicly available genome sequences were selected for comparison with DD3^T^. The ANI and dDDH values obtained for the type strains of the respective species are listed in Table 1, where the data are presented in a descending order of their ANIb values calculated against the DD3^T^ genome sequence.

The ANIb values between DD3^T^ and its closely related species ranged from 78.20% (*Bradyrhizobium cenepequi* CNPSo 4026^T^) to 79.92% (*A. broomeae* ATCC 49717^T^), whereas the OrthoANIu values ranged from 74.44% (*Bradyrhizobium canariense* BTA-1^T^) to 78.06% (*A. massiliensis* DSM 17498^T^). Both ANI values were below the cut-off value for prokaryotic species delineation [18]. The dDDH values between DD3^T^ and its closely related species ranged from 20.1% (*Bradyrhizobium betae* PL7HG1^T^) to 21.5% (*A. broomeae* ATCC 49717^T^), which were lower than the threshold for prokaryotic species delineation [18].

To further determine the phylogenetic position of DD3^T^/DDX28, a comprehensive phylogenomic analysis was conducted based on the concatenated alignment of core genes, which constitute the core genome of DD3^T^/DDX28, the closely related 41 species (Table 1) and *Pseudorhodoplanes sinuspersici* (outgroup). Genomic annotations were performed *de novo* using Prokka version 1.14.6 [29], and the annotated genes were compared among the input genomes using the Roary pan-genome analysis pipeline version 3.13.0 [30], with an amino acid identity threshold of 70%. This analysis revealed 475 core genes present in all tested genomes. Subsequently, nucleotide sequences of the core genes were concatenated, and multiple alignments were performed using MAFFT version 7.475 [31], which was implemented through Roary. Poorly aligned positions and divergent regions were eliminated from the concatenated alignment using Gblocks version 0.91b [32] with default parameters. From the resulting alignment (total alignment length of 334 305 bp), a maximum-likelihood tree was reconstructed using RAxML-NG version 1.2.1 [33] with a general time-reversible substitution model and a gamma model of rate heterogeneity. To assess the reliability of the tree, the standard bootstrap method with 100 replicates was used. The resulting phylogenomic tree (Fig. 1) placed DD3^T^/DDX28 and *Pseudomonas carboxydohydrogena* within the clade of the genus *Afipia*. However, it is noteworthy that the phylogenetic position of DD3^T^/DDX28 did not match that of any other member of this clade.

## Physiology and chemotaxonomy

Phenotypic characteristics of DD3^T^/DDX28 were compared with those of the type strains of six *Afipia* species (*A. broomeae*, *A. massiliensis*, *A. birgiae*, *A. clevelandensis*, *A. felis* and *A. carboxidovorans*) exhibiting the highest ANIb values against DD3^T^ (Table 1). Data of *A. broomeae*, *A. massiliensis*, *A. birgiae*, *A. clevelandensis* and *A. felis* were retrieved from the study of La Scola *et al*. [5] for comparison. DD3^T^/DDX28 and *A. carboxidovorans* DSM 1227^T^ were cultured routinely at 30 °C on standard methods agar (SMA) plates (Nissui), and their phenotypic characteristics were evaluated. Growth was tested at 30 °C on BCYE agar, MacConkey agar (Nissui), SMA, nutrient broth (Difco) and 1% (w/v) peptone water alone or with 6% (w/v) NaCl. Growth at different temperatures (25, 30, 35, 37 and 42 °C) was evaluated using BCYE agar plates. Cell size and morphology were assessed via transmission electron microscopy, as previously described [21]. The presence of flagella was examined via Leifson staining [34]. Motility was assessed with semi-solid SMA medium [0.4% (w/v) agar] using the stabbing technique [35] and confirmed using the hanging drop method [36]. Gram reaction and oxidase activity were tested using the protocols described by Schaad *et al*. [35]. Catalase activity was determined as described by Lelliott *et al*. [37]. Additionally, biochemical and physiological characteristics were determined in triplicate using the API 20 NE kit (bioMérieux), according to the manufacturer’s protocol, with some modifications. Briefly, the bacterial concentration of the inoculum (saline suspension) used to inoculate the strips was adjusted to ~10^9^ c.f.u. ml^−1^, and the results of tests were scored after 7 days of incubation at 30 °C.

**Fig. 2.**
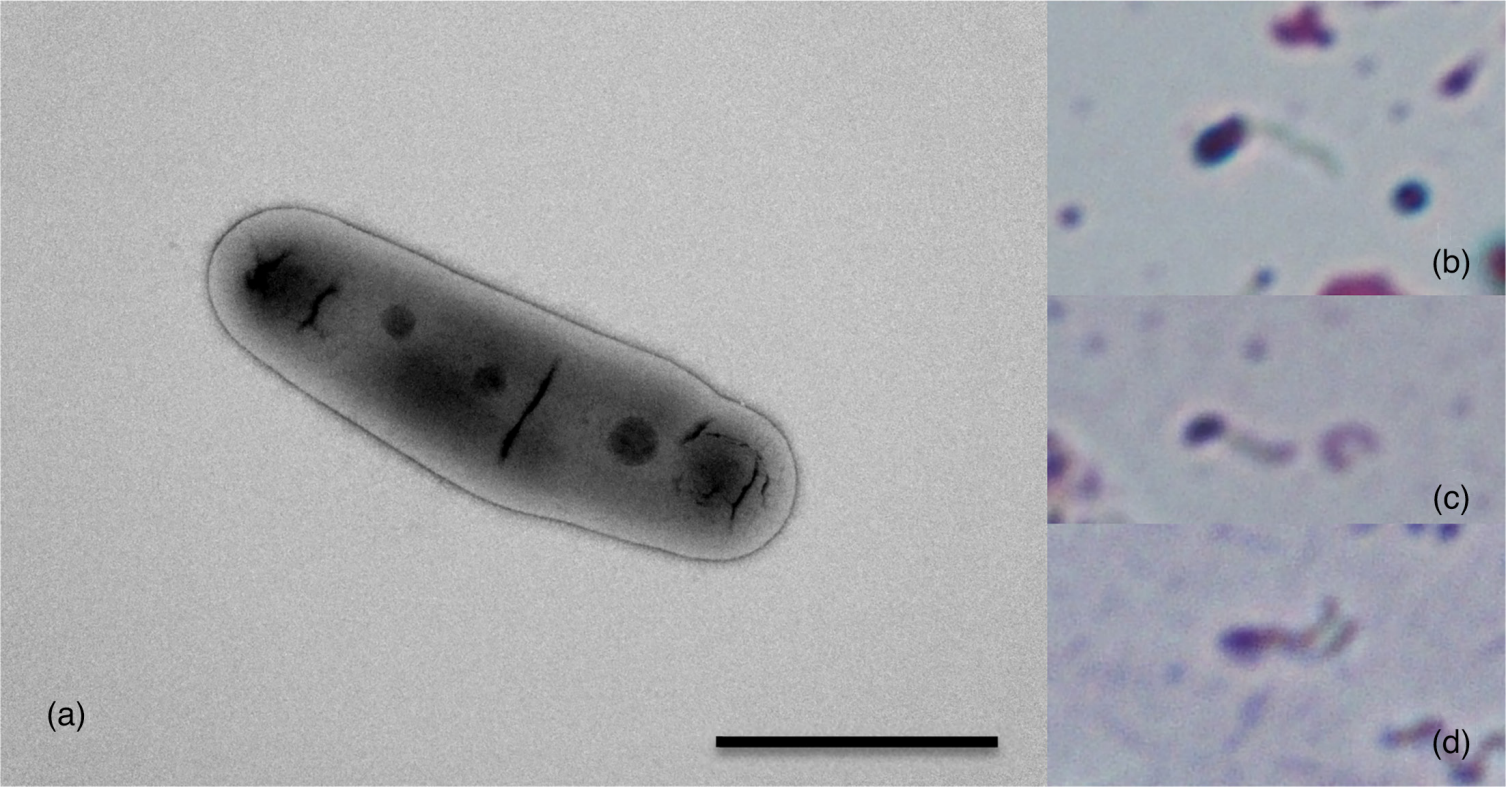
Transmission electron micrograph (**a**) and light micrographs (b–d) of *A. dichlorophenoxyacetatis* sp. nov. strain DD3^T^, showing rod-shaped cells (a–d) and one or two polar flagella (b–d). Scale bar, 1 µm.

Cells of DD3^T^/DDX28 were rod-shaped, often lacking flagella, but some showed motility due to the presence of one or two polar flagella (Fig. 2). The cell size (mean±sd) of DD3^T^ was 2.1±0.5 µm×0.7±0.03 µm (*n*=13). DD3^T^/DDX28 grew on BCYE agar, SMA and peptone water; grew weakly on nutrient broth; but failed to grow on MacConkey agar or peptone water containing 6% NaCl. Growth of DD3^T^/DDX28 was observed at 25 and 30 °C, but not at ≥35 °C. They exhibited slow growth; on BCYE agar plates, DD3^T^/DDX28 formed visible colonies after incubation for 5 days at 30 °C. The diameter of colonies was ~0.5–1.5 mm after around 7 days of incubation. The colonies formed on BCYE agar plates were white to greyish-white in colour, opaque, round with entire margins, convex, smooth and glistening (Fig. S3). No diffusible pigments were observed when cultured on the plates. DD3^T^/DDX28 were Gram-reaction-negative and aerobic and exhibited a positive response to oxidase and a weakly positive response to catalase. In API 20 NE tests, the strains were positive for nitrate reduction, urease and assimilation of potassium gluconate, but negative for indole production, glucose fermentation, arginine dihydrolase, aesculin hydrolysis, gelatin hydrolysis, *β*-galactosidase and assimilation of d-glucose, l-arabinose, d-mannose, d-mannitol, *N*-acetyl-d-glucosamine, maltose, capric acid, adipic acid, malic acid, trisodium citrate and phenylacetic acid.

Differences in phenotypic characteristics between DD3^T^/DDX28 and the closest relatives are summarized in Table 2. DD3^T^/DDX28 was distinguished from the closest relatives based on the following characteristics: positive for motility, nitrate reduction and assimilation of potassium gluconate and negative for growth on MacConkey agar, growth at ≥35 °C on BCYE agar, arginine dihydrolase and assimilation of adipic acid, malic acid and phenylacetic acid. Detailed phenotypic characteristics of DD3^T^/DDX28 are provided in the description of the species.

**Table 2.** Characteristics that differentiate *A. dichlorophenoxyacetatis* sp. nov. strains from the type strains of the most closely related species Strains: 1–1, *A. dichlorophenoxyacetatis* DD3^T^; 1–2, *A. dichlorophenoxyacetatis* DDX28; 2, *A. broomeae* B-91-007286^T^; 3, *A. massiliensis* 34633^T^; 4, *A. birgiae* 34632^T^; 5, *A. clevelandensis* B-91-007353^T^; 6, *A. felis* B-91-007352^T^; 7, *A. carboxidovorans* DSM 1227^T^. Data of *A. dichlorophenoxyacetatis* and *A. carboxidovorans* were obtained in this study. The other data were retrieved from the study of La Scola *et al* [5]. Characteristics have been scored as follows: +, positive reaction; −, negative reaction; w, weakly positive reaction.

Characteristic	1–1	1–2	2	3	4	5	6	7
Motility	+	+	+	+	−	+	+	+
Growth on MacConkey agar	−	−	−	−	−	w	−	w
Growth on BCYE agar at:								
35 °C	−	−	+	−	−	w	+	+
37 °C	−	−	+	−	−	−	+	+
API 20 NE test*:								
Nitrate reduction	+	+	−	+	+	−	+	−
Arginine dihydrolase	−	−	−	−	−	−	−	+
Assimilation of(API 20 NE)*:								
Potassium gluconate	+	+	+	−	−	+	−	−
Adipic acid	−	−	+	+	+	+	−	−
Malic acid	−	−	+	−	−	+	+	+
Phenylacetic acid	−	−	+	−	−	−	−	−
Isolation source	Field soil	Field soil	Human sputum	Water supply of hospital	Water supply of hospital	Human tibia	Human lymph node tissue	Wastewater
Genome size (Mb)†	6.0	6.0	5.3	5.3	5.3	4.4	4.2	3.7
DNA G+C content (mol%)†	58.8	58.8	61.3	60.9	60.8	61.7	60.7	62.4

* The results were scored after incubation for 7 days at 30 °C.

† These results were calculated from the respective genomic DNA sequences.

For cellular fatty acid analysis, DD3^T^ and *A. carboxidovorans* DSM 1227^T^ were cultured on BCYE agar plates at 30 °C for 4 days. Cellular fatty acids were analysed using the Sherlock Microbial Identification System version 6.0 (MIDI) and CLIN6 method, according to our previous studies [21,22]. The major fatty acids (>10 % of the total fatty acids) in DD3^T^ (Table 3) were summed feature 8 (52.1 %), C_18 : 0_ (13.4%) and C_17 : 0_ cyclo (10.1%). The presence of summed feature 3, summed feature 8, C_18 : 1_* ω7c* 11-methyl, C_17 : 0_ cyclo and C_18 : 0_ in DD3^T^ was consistent with the reported fatty acid profiles of the *Afipia* species [4]. Differences in the presence/absence of the following fatty acid components were observed between DD3^T^ and the type strains of its closest relatives (Table 3): C_15 : 0_, C_17 : 0_, C_17 : 0_ anteiso DMA (dimethyl acetal), C_19 : 0_, C_12 : 0_ 3OH, C_17 : 0_ iso 2OH, C_17 : 1_* ω8c* and C_20 : 1_* ω7c*.

**Table 3.** Cellular fatty acid composition (%) of *A. dichlorophenoxyacetatis* sp. nov. strain DD3^T^ and the type strains of the most closely related species Strains: 1, *A. dichlorophenoxyacetatis* DD3^T^; 2, *A. broomeae* CCUG 30458^T^; 3, *A. massiliensis* CCUG 45153^T^; 4, *A. birgiae* CCUG 43108^T^; 5, *A. clevelandensis* CCUG 30457^T^; 6, *A. felis* CCUG 30456^T^; 7, *A. carboxidovorans* DSM 1227^T^. Data of *A. dichlorophenoxyacetatis* and *A. carboxidovorans* were obtained in this study. The other data were retrieved from the Culture Collection University of Gothenburg (CCUG) (https://www.ccug.se). TR, trace amount (<1 %); –, not detected. Fatty acids representing <1% in all strains are not shown. The major fatty acids of each species (>10% of the total fatty acids) are highlighted in bold.

Fatty acid	1	2	3	4	5	6	7
C_15 : 0_	–	tr	–	1.8	–	–	–
C_16 : 0_	2.9	5.2	3.6	3.1	5.5	**13.9**	1.9
C_17 : 0_	tr	3.5	–	7.1	tr	–	–
C_17 : 0_ anteiso DMA	–	1.7	–	–	–	–	–
C_18 : 0_	**13.4**	**15.7**	**11.3**	5.1	**12.2**	5.9	**11.7**
C_19 : 0_	–	1.0	–	–	–	–	–
C_12 : 0_ 3OH	tr	–	–	–	–	–	2.6
C_17 : 0_ iso 2OH	–	–	–	3.7	–	–	–
C_17 : 0_ cyclo	**10.1**	**19.9**	**27.1**	**27.1**	**23.1**	**20.5**	**11.0**
C_19 : 0_ cyclo *ω*8*c*	3.3	**15.4**	8.6	3.0	8.6	**24.1**	**24.8**
C_17 : 1_* ω8c*	tr	–	–	7.1	–	–	–
C_18 : 1_* ω7c* 11-methyl	7.6	**12.8**	**14.1**	7.8	**13.3**	8.1	tr
C_20 : 1_ *ω*7*c*	1.4	–	tr	–	tr	–	tr
Summed feature 3*	7.2	4.8	9.5	**15.3**	9.2	5.7	4.8
Summed feature 8*	**52.1**	**14.6**	**24.3**	**16.2**	**26.6**	**21.7**	**39.7**

* Summed features are fatty acids that cannot be resolved reliably from another fatty acid using the chromatographic conditions chosen. The MIDI system groups these fatty acids together as one feature with a single percentage of the total. Summed feature three comprises C_16 : 1_* ω7c* and/or C_16 : 1_* ω6c*. Summed feature eight comprises C_18 : 1_* ω7c* and/or C_18 : 1_* ω6c*.

## Functional genomics

In DD3^T^, *cad* and *tfd* gene clusters accumulate as a single gene cluster constituting 2,4-D degradation genes [12]. Here, *cadA* (encoding a subunit of 2,4-D oxygenase) and *tfdC* (encoding chlorocatechol 1,2-dioxygenase) were selected as representatives of the *cad* and *tfd* gene clusters, respectively. Genome blast Service of EzBioCloud and Microbial Nucleotide blast of NCBI were used to determine whether there are regions of high homology to the sequences of these two genes of DD3^T^ in the genomes of the closest relatives (the six known species of the genus *Afipia*; Table 1). blast searches using the *cadA* or *tfdC* sequences of DD3^T^ as queries did not reveal any regions of high homology to these in the genomes of the closest relatives. These results, together with previous reports of 2,4-D degradation genes being acquired via horizontal transfer in other bacteria [9,11], suggest that the presence of 2,4-D degradation genes in DD3^T^ may also be due to horizontal transfer.

Functional annotation and metabolic reconstruction of the genomes of DD3^T^ and its closest relatives were performed using BlastKOALA version 3.0 and KEGG Mapper version 5.1 in the Kyoto Encyclopedia of Genes and Genomes (KEGG) (https://www.kegg.jp/kegg/) [38]. The outputs of BlastKOALA were further parsed and visualized using KEGG Decoder version 1.3 [39] to assess the completeness or presence/absence of major metabolic processes, including metabolic pathways, multi-subunit proteins and singular proteins. Furthermore, the genomes of DD3^T^ and its closest relatives were searched for the presence of secondary/specialized metabolite biosynthetic gene clusters (BGCs) using antiSMASH bacterial version 7.0.0 [40] (https://antismash.secondarymetabolites.org/) with the detection strictness level set as ‘relaxed’.

KEGG annotation predicted that the DD3^T^ genome encodes 67 complete pathway modules, which were organized into the following functional subcategories in the metabolism category: carbohydrate metabolism, energy metabolism, lipid metabolism, nucleotide metabolism, amino acid metabolism, glycan metabolism, metabolism of cofactors and vitamins and biosynthesis of terpenoids and polyketides. In terms of the number of genes assigned to each functional subcategory, genes for carbohydrate metabolism (293 genes) and amino acid metabolism (250) were the most abundant in the DD3^T^ genome (Fig. S4). The DD3^T^ genome was predicted to incompletely encode the arginine dihydrolase (deiminase) pathway for arginine degradation under oxygen-limiting conditions [41], which consists of the following three enzymes: arginine deiminase (EC 3.5.3.6), catabolic ornithine carbamoyltransferase (EC 2.1.3.3) and carbamate kinase (EC 2.7.2.2). Among them, DD3^T^ harboured the gene encoding catabolic ornithine carbamoyltransferase, but not those encoding the other two enzymes. This annotation result was consistent with the experimental result (Table 2). In contrast, DD3^T^ was predicted to possess the complete pathway module required for assimilatory nitrate reduction (KEGG Module: M00531) [42], which was experimentally confirmed (Table 2). Genes encoding flagellar biosynthesis and assembly proteins, including *flgABCDEFGHIJKL*, *flhAB*, *fliCDEFGHIJLMNPQRS* and *motAB*, were predicted to be present in the DD3^T^ genome, which is consistent with the result that some cells of DD3^T^ exhibit motility due to the presence of one or two polar flagella (Table 2, Fig. 2). Additionally, many genes potentially involved in membrane transport (including ABC transporters and bacterial secretion system) (118 genes), signal transduction (including two-component system) (108), cellular community (including quorum sensing and biofilm formation) (75) and bacterial chemotaxis (12) were detected, suggesting that DD3^T^ may be sensitive to various environmental stimuli.

Comparisons of the distribution patterns of genes in the functional subcategories between DD3^T^ and its closest relatives revealed consistent rough trends, with minor differences (Fig. S4). DD3^T^ harboured more genes associated with carbohydrate metabolism and metabolism of cofactors and vitamins than its closest relatives. Moreover, the number of genes predicted to be involved in membrane transport, signal transduction and cellular community was slightly higher in DD3^T^ than in its closest relatives.

**Fig. 3.**
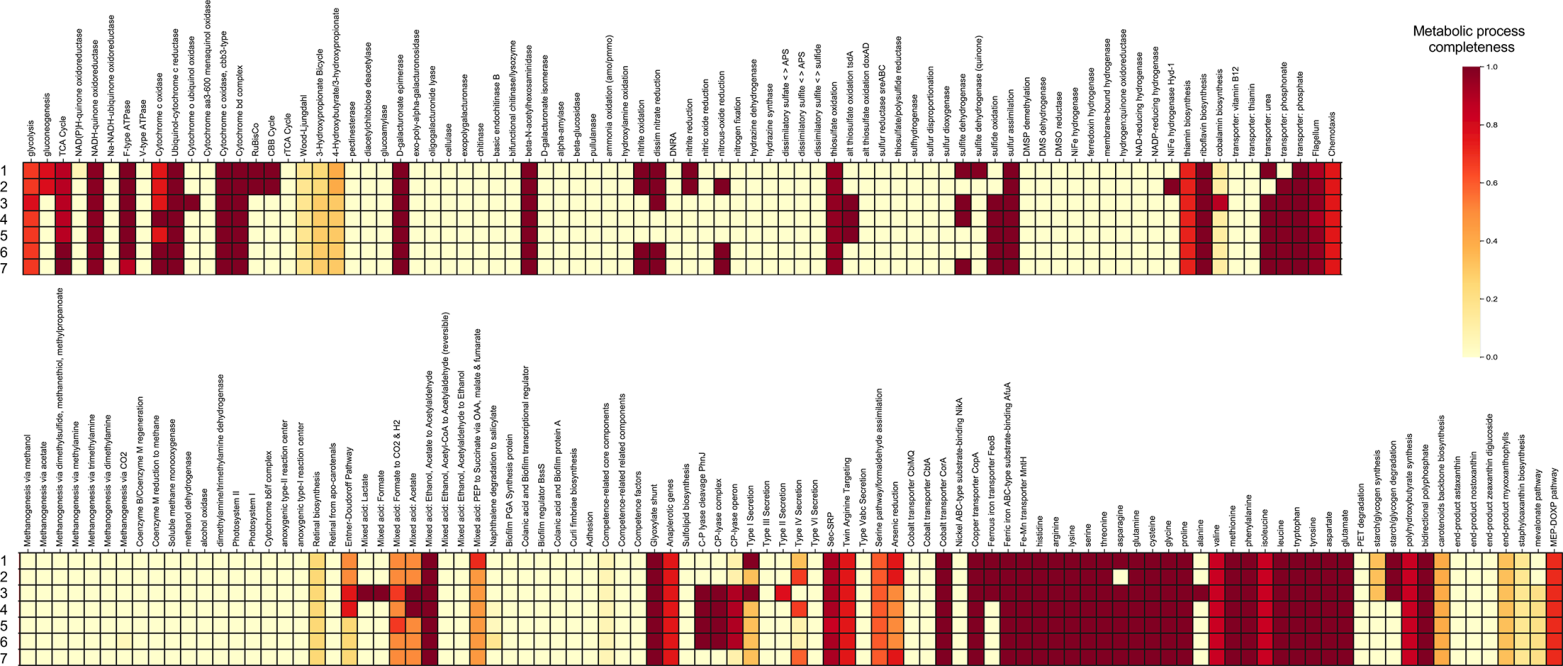
KEGG Decoder heat map based on BlastKOALA annotations. The heat map shows the metabolic process completeness in *A. dichlorophenoxyacetatis* DD3^T^ and its closest relatives based on the presence or absence of genes inferred by BlastKOALA [38] and KEGG Decoder [39]. Metabolic processes are represented on a scale from 0 to 1 denoting the fraction of completeness that a process has within a genome. Strains: 1, *A. felis* ATCC 53690^T^; 2, *A. carboxidovorans* OM5^T^; 3, *A. dichlorophenoxyacetatis* DD3^T^; 4, *A. broomeae* ATCC 49717^T^; 5, *A. clevelandensis* ATCC 49720^T^; 6, *A. massiliensis* DSM 17498^T^; 7, *A. birgiae* 34632^T^

Further comparisons of their genomic contents via BlastKOALA and KEGG Decoder analyses revealed differences between DD3^T^ and its closest relatives regarding the completeness and presence/absence of metabolic processes (Fig. 3). The following processes showed particularly marked differences with respect to their completeness: cytochrome o ubiquinol oxidase, Calvin cycle, nitrite oxidation, nitrate reductase (cytochrome), nitrite reductase (NO-forming), nitrous-oxide reductase, alternative thiosulfate oxidation (*tsdA*), sulfite dehydrogenase (cytochrome), sulfite dehydrogenase (quinone), sulfide oxidation, [NiFe] hydrogenase Hyd-1, urea transport system, phosphonate transport system, lactate fermentation, formate fermentation, C–P lyase complex, ferrous iron transport protein B, asparagine metabolism, alanine metabolism and starch/glycogen degradation. However, it remains unclear whether these processes are functional, how the presence/absence of these processes affects each strain and what role these processes play in the ecology of each strain, and further research is needed.

AntiSMASH results indicated that the DD3^T^ genome harboured five putative BGCs responsible for secondary metabolite production, which showed low-level similarity (15%) or non-similarity to known BGCs (Table S5). A comparison of the distribution patterns of putative BGCs between DD3^T^ and its closest relatives revealed that these patterns were broadly similar, with some minor differences (Table S6). The following types of BGCs were detected in all or most genomes analysed: terpene, redox-cofactor and homoserine lactone-type BGCs. In contrast, the following types of BGCs were observed only in one or some genomes: *N*-acyl amino acids (acyl amino acids), linear azol(in)e-containing peptide, non-ribosomal peptide synthetase, unspecified ribosomally synthesized and post-translationally modified peptide (RiPP-like), RiPP-recognition-element-containing cluster and lasso peptide (lassopeptide)-type BGCs. However, most of the above-mentioned putative BGCs exhibited no homology to known BGCs, suggesting that *Afipia* species may have high potential for synthesizing novel natural products. These results indicate that future studies are needed to determine whether the commonly found BGCs contribute to the synapomorphy of *Afipia* species and whether the specificity of a BGC found only in a specific strain contributes to the properties of that strain (Table S6). With further research, the species-specific metabolic processes and BGCs identified here (Fig. 3, Table S6) could be used as promising indicators for the classification and identification of *Afipia* species.

## Taxonomic conclusion

Phylogenetic analyses based on the *gyrB* gene sequences (Fig. S2) and examination of the cellular fatty acid composition (Table 3), as well as the preliminary genome analyses using TYGS and DFAST (Tables S2 and S3), confirmed that DD3^T^/DDX28 belong to the genus *Afipia*. Phylogenomic analysis using the whole-genome sequences revealed that the phylogenetic position of the strains does not match those of any known species in the genus (Fig. 1). ANIb, OrthoANIu and dDDH results (Table 1) were consistent with the phylogenomic findings (Fig. 1), indicating that DD3^T^/DDX28 constitute a novel species of the genus *Afipia*, for which we propose the name *A. dichlorophenoxyacetatis* sp. nov., with DD3^T^ (MAFF 311804^T^=ICMP 25015^T^) as the type strain. *A. dichlorophenoxyacetatis* sp. nov. can be differentiated from its closest relatives based on its phenotypic characteristics (Table 2), cellular fatty acid composition (Table 3) and species-specific metabolic processes (including 2,4-D degradation) and BGCs (Fig. 3, Table S6).

Based on the results of this study (Figs 1, S1 and S2), *Pseudomonas carboxydohydrogena* should be transferred to the genus *Afipia*; however, this needs to be validated in future taxonomic studies. Furthermore, a number of genome sequences have been reported for the genus *Afipia*, including those related to ‘*Afipia septicemium*’, ‘*Afipia cberi*’ and ‘*Candidatus* Afipia apatlaconensis’ [43,45]. Therefore, it will be important to conduct comprehensive comparative genomic and phylogenomic analyses, including these, to clarify the actual diversity surrounding the genus *Afipia* and then organize it taxonomically.

## Description of *Afipia dichlorophenoxyacetatis* sp. nov.

*Afipia dichlorophenoxyacetatis* (di.chlo.ro.phen.o.xy.a.ce.ta'tis. N.L. gen. n. *dichlorophenoxyacetatis*, pertaining to dichlorophenoxyacetate).

Members of this species are Gram-reaction-negative, oxidase-positive, weakly catalase-positive, aerobic and non-spore-forming. Cells are rod-shaped and often lack flagella, but some exhibit motility owing to the presence of one or two polar flagella. The cell size (mean±sd) is 2.1±0.5 µm×0.7±0.03 µm (*n*=13). It grows on BCYE agar, SMA (plate count agar) and 1% (w/v) peptone water; grows weakly on nutrient broth; but fails to grow on MacConkey agar or 1% (w/v) peptone water containing 6% (w/v) NaCl. Growth is observed at 25 and 30 °C but not at ≥35 °C on BCYE agar plates. Colonies are white to greyish-white in colour, opaque, round with entire margins, convex, smooth and glistening, with a diameter of ~0.5–1.5 mm, after culturing on BCYE agar plates for 7 days at 30 °C. No diffusible pigments are observed when cultured on the plates.

In API 20 NE tests (7 days incubation at 30 °C), they are positive for nitrate reduction, urease and assimilation of potassium gluconate but negative for indole production, glucose fermentation, arginine dihydrolase, aesculin hydrolysis, gelatin hydrolysis, *β*-galactosidase and assimilation of d-glucose, l-arabinose, d-mannose, d-mannitol, *N*-acetyl-d-glucosamine, maltose, capric acid, adipic acid, malic acid, trisodium citrate and phenylacetic acid.

The major cellular fatty acids (>10% of the total fatty acids) in DD3^T^ are summed feature 8 (C_18 : 1_* ω7c* and/or C_18 : 1_* ω6c*), C_18 : 0_ and C_17 : 0_ cyclo. The genomic DNA G+C content of DD3^T^ is 58.8 mol%, and its genomic size is ~5.96 Mb.

The type strain, DD3^T^ (MAFF 311804^T^=ICMP 25015^T^), was isolated from field soil in Ibaraki Prefecture, Japan, in 2005. It has the ability to utilize 2,4-D as the sole carbon source. DDX28 (MAFF 311814=ICMP 25016) is an additional strain belonging to this species.

The genome sequencing data of DD3^T^ and DDX28 have been deposited in DDBJ/ENA/GenBank under accession numbers AP029250 and AP029255, respectively. The 16S rRNA gene sequences of DD3^T^ and DDX28 are available under accession numbers LC770309 and LC770310, respectively.

## Supplementary material

10.1099/ijsem.0.006672 Uncited Supplementary Material 1.

## Abbreviations

ANI: average nucleotide identity
ANIb: average nucleotide identity algorithm using BLAST
BCYE: buffered charcoal-yeast extract
BGC: biosynthetic gene cluster
2,4-D: 2,4-dichlorophenoxyacetic acid
dDDH: digital DNA–DNA hybridization
DFAST: DDBJ fast annotation and submission tool
GGDC3: genome-to-genome distance calculator 3.0
KEGG: kyoto encyclopedia of genes and genomes
OrthoANIu: orthologous ANI algorithm using USEARCH
RiPP: ribosomally synthesized and post-translationally modified peptide
SMA: standard methods agar
TYGS: type strain genome server
