# Supplementary material for: Afipia dichlorophenoxyacetatis sp. nov., isolated from field soil in Japan, degrades 2,4-dichlorophenoxyacetic acid
Source: Int J Syst Evol Microbiol. 2025 Feb 10;75(2):006672. doi: 10.1099/ijsem.0.006672 (PMC12282010; doi:10.1099/ijsem.0.006672)
Supplement: Uncited Supplementary Material 1. [file ijsem-75-06672-s001.pdf]

IJSEM supplementary materials

Afipia dichlorophenoxyacetatis sp. nov., isolated from field soil in Japan, degrades 2,4-dichlorophenoxyacetic acid

Hiroyuki Sawada[1*], Yoriko Sakai[2], Yusuke Takashima[1], Ken Naito[1], Mitsuo Horita[2] and Mamoru Satou[1]

Author affiliations:

[1] Research Center of Genetic Resources, National Agriculture and Food Research Organization (NARO), 2-1-2 Kannondai, Tsukuba, Ibaraki 305-8602, Japan

[2] Institute for Agro-Environmental Sciences, NARO, 3-1-3 Kannondai, Tsukuba, Ibaraki 305-8604, Japan

*** Correspondence:**

Hiroyuki Sawada, sawada@naro.affrc.go.jp

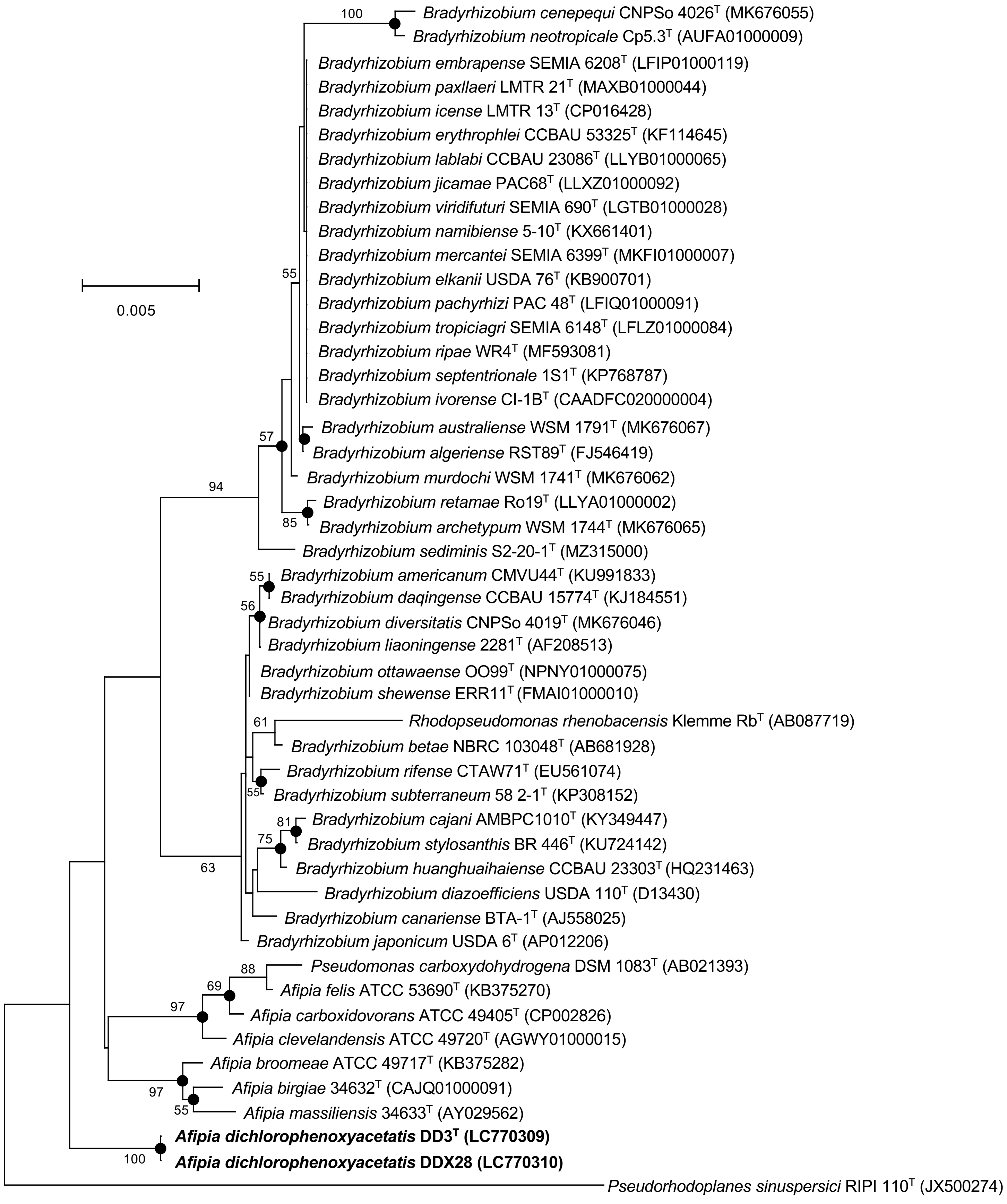

Fig. S1. Neighbour-joining tree based on the 16S rRNA gene sequences, showing the relationships between *Afipia dichlorophenoxyacetatis* sp. nov. strains (boldface type) and closely related species (Table S4). *Pseudorhodoplanes sinuspersici* RIPI 110^T was used as an outgroup. Evolutionary distances were computed using the maximum composite likelihood method. Rate variation among sites was modelled with gamma distribution (shape parameter = 1). T, type strain of the species. Numbers at nodes indicate the standard bootstrap values (≥ 50%) from 1000 repetitions. Filled circles indicate that the corresponding nodes were also recovered in the maximum-likelihood and maximum-parsimony trees. Bar, 0.005 substitutions per nucleotide position.

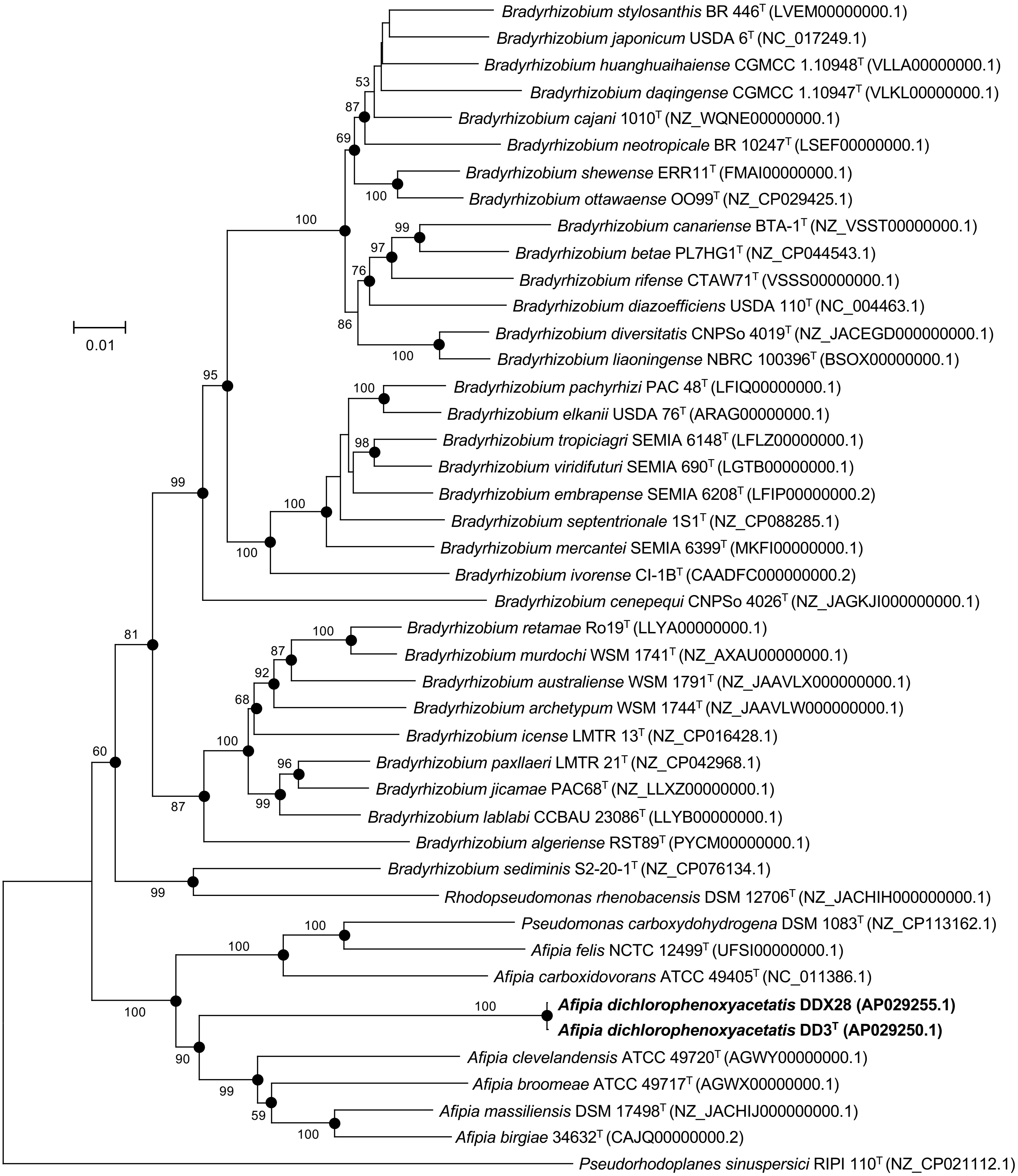

Fig. S2. Neighbour-joining tree based on the *gyrB* gene sequences, showing the relationships between *Afipia dichlorophenoxyacetatis* sp. nov. strains (boldface type) and closely related species (Table 1). *Pseudorhodoplanes sinuspersici* RIPI 110^T was used as an outgroup. The sequences used here were extracted from the respective whole-genome sequences (accession numbers are shown in parentheses). Evolutionary distances were computed using the maximum composite likelihood method. Rate variation among sites was modelled with gamma distribution (shape parameter = 1). T, type strain of the species. Numbers at nodes indicate the standard bootstrap values (≥ 50%) from 1000 repetitions. Filled circles indicate that the corresponding nodes were also recovered in the maximum-likelihood and maximum-parsimony trees. Bar, 0.01 substitutions per nucleotide position.

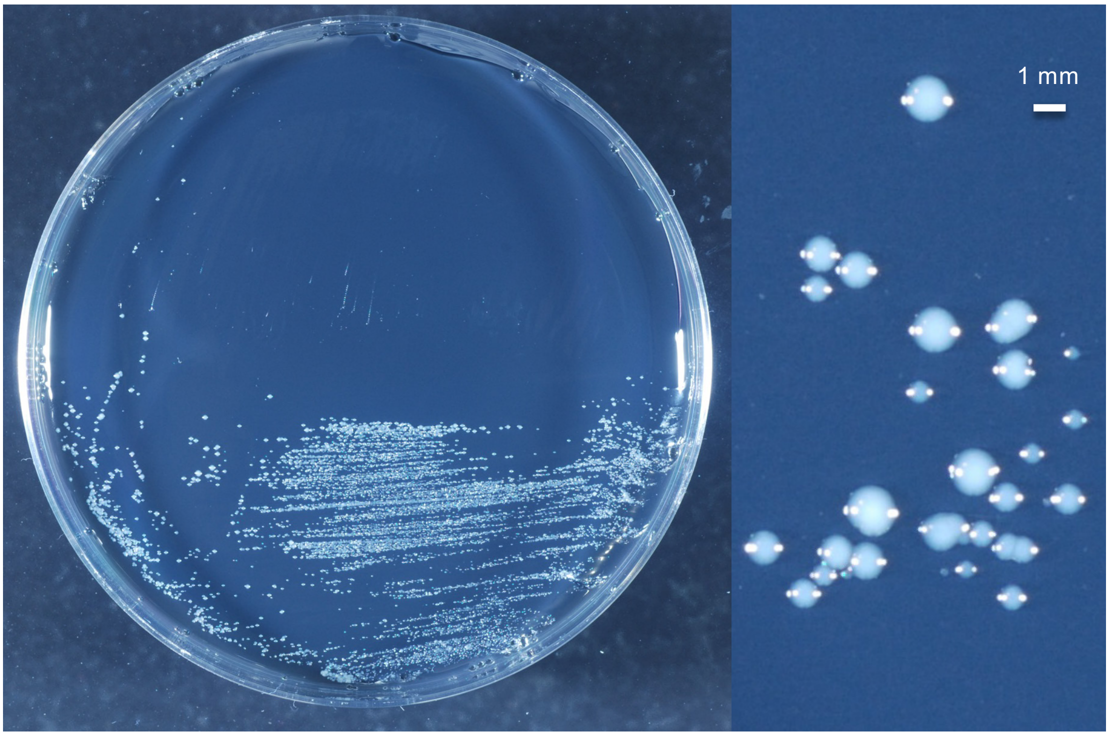

Fig. S3. Colony colour and morphology of *Afipia dichlorophenoxyacetatis* sp. nov. strain DD3^{T} on a buffered charcoal-yeast extract (BCYE) agar plate incubated at 30 °C for seven days.

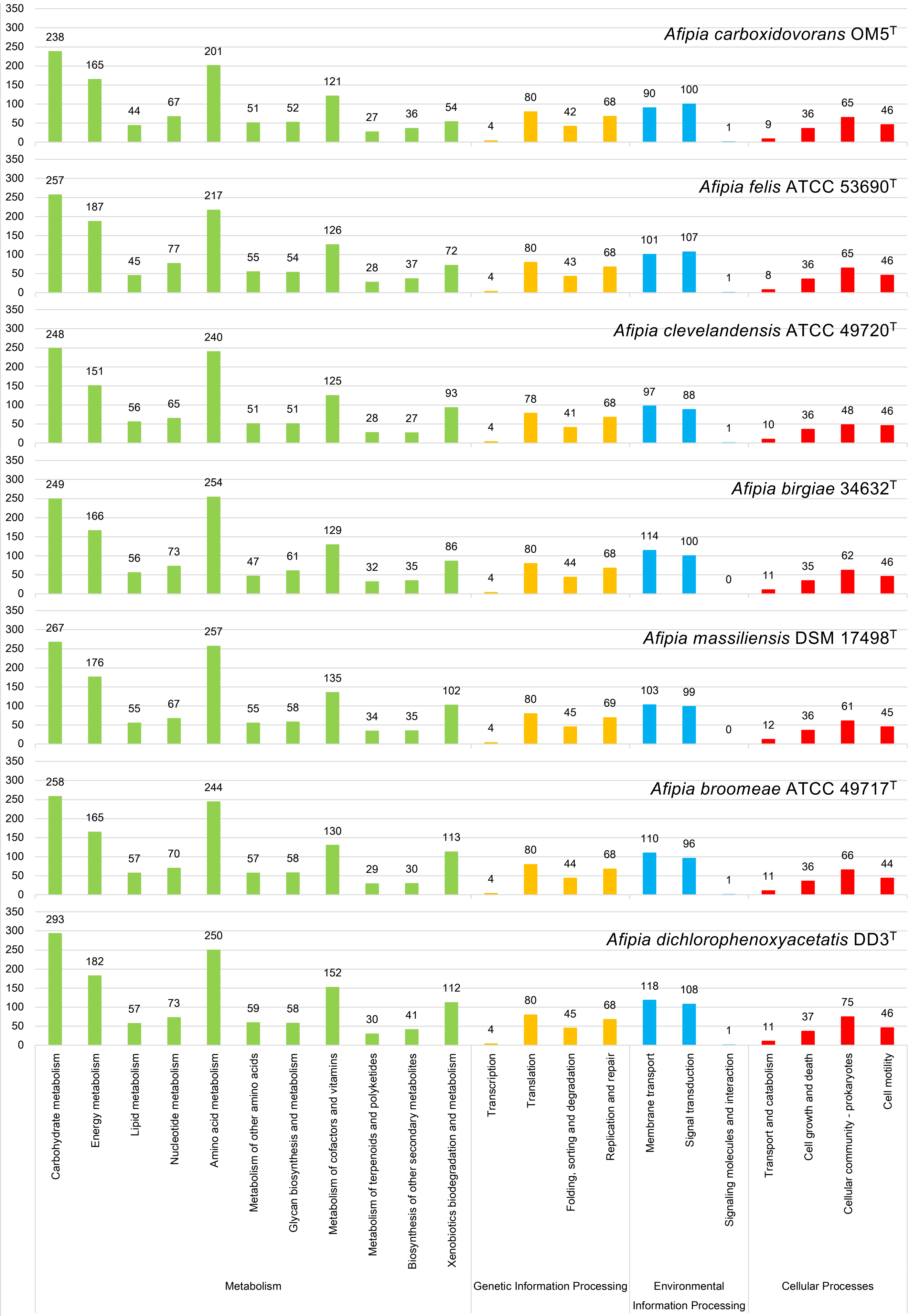

Fig. S4. Numbers of genes in the genomes of *Afipia dichlorophenoxyacetatis* DD3^T and its closest relatives assigned to each KEGG-defined function using the BlastKOALA tool. X and Y axes show the 22 subcategories distributed in the four major categories and number of genes assigned to each subcategory, respectively.

Table S1. Characteristics of the genome sequences

Characteristic	$DD3^T$ *	DDX28
Total no. of contigs:		
Chromosome	1	1
Plasmid	0	0
Circular	Yes	Yes
DDBJ/ENA/GenBank Nucleotide accession number	AP029250	AP029255
Sequence Read Archive accession number	NA	DRR530028
Size (bp)	5,959,066	5,959,441
DNA G+C content (mol%)	58.8	58.8
No. of CDSs	5,686	5,876
No. of rRNA genes	2, 2, 2 (5S, 16S, 23S)	2, 2, 2 (5S, 16S, 23S)
No. of tRNA genes	51	51
No. of CRISPRs	0	0
Coding ratio (%)	86.9	86.3
Completeness (%) †	99.46	98.40
Contamination (%) †	0.91	0.91

* The whole–genome sequence of $DD3^T$ was determined and described in the previous study [12].

† The quality of the genome sequences was assessed using CheckM [Parks et al., 2015].

Parks DH, Imelfort M, Skennerton CT, Hugenholtz P, Tyson GW. CheckM: assessing the quality of microbial genomes recovered from isolates, single cells, and metagenomes. *Genome Res.* 2015;25:1043–1055.

Table S2. Pairwise comparisons of the DD3^T and type strain genomes using TYGS

TYGS (Type Strain Genome Server) [19] is available at https://tygs.dsmz.de. The data shown here were calculated with Formula d_4 (d_4 is identical to GGDC Formula 2) using the DD3^T genome sequence as a query and are presented in a descending order of their dDDH values.

**Species**	**Strain**	**dDDH (d_4, in %)**	**Confidence intervals (d_4, in %)**
Afipia broomeae	ATCC 49717^T	21.5	[19.2 - 23.9]
Afipia massiliensis	DSM 17498^T	21.5	[19.2 - 23.9]
Afipia clevelandensis	ATCC 49720^T	21.2	[19.0 - 23.7]
Bradyrhizobium jicamae	PAC68^T	21.1	[18.9 - 23.5]
"Bradyrhizobium oropedii"	Pear76^T	21.0	[18.8 - 23.5]
Bradyrhizobium mercantei	SEMIA 6399^T	20.9	[18.7 - 23.4]
Bradyrhizobium paxllaeri	LMTR 21^T	20.9	[18.7 - 23.3]
Bradyrhizobium icense	LMTR 13^T	20.8	[18.6 - 23.2]
Bradyrhizobium septentrionale	1S1^T	20.8	[18.6 - 23.2]
"Bradyrhizobium macuxiense"	BR 1-0303^T	20.8	[18.6 - 23.2]
Bradyrhizobium embrapense	SEMIA 6208^T	20.8	[18.5 - 23.2]
Bradyrhizobium viridifuturi	SEMIA 690^T	20.7	[18.5 - 23.1]
Bradyrhizobium diazoefficiens	USDA 110^T	20.6	[18.4 - 23.1]
Bradyrhizobium sediminis	S2-20-1^T	20.6	[18.4 - 23.0]
Bradyrhizobium australiense	WSM 1791^T	20.6	[18.3 - 23.0]
"Rhodopseudomonas infernalis"	HC1^T	20.5	[18.3 - 22.9]
Bradyrhizobium rifense	CTAW71^T	20.4	[18.2 - 22.8]
Bradyrhizobium cenepequi	CNPSo 4026^T	20.3	[18.1 - 22.8]

Table S3. Pairwise comparisons of the DD3^{T} and type strain genomes based on Taxonomy Check implemented in DFAST

DFAST (DDBJ Fast Annotation and Submission Tool) [15] is available at https://dfast.ddbj.nig.ac.jp. The data shown here were calculated with the FastANI algorithm [20] using the DD3^{T} genome sequence as a query and are presented in a descending order of their ANI values.

Species	Strain	Accession	Taxonomy ID	ANI (%)	Matched fragments	Total fragments	Status
Afipia broomeae	ATCC 49717^{T}	GCA_000314675.2	56946	80.036	818	1986	below_threshold
Afipia massiliensis	DSM 17498^{T}	GCA_014203115.1	211460	80.034	782	1986	below_threshold
Afipia birgiae	34632^{T}	GCA_000308295.2	151414	79.901	764	1986	below_threshold
Afipia clevelandensis	ATCC 49720^{T}	GCA_000336555.1	1034	79.725	748	1986	below_threshold
Afipia felis	ATCC 53690^{T}	GCA_000314735.2	1035	78.973	449	1986	below_threshold
Afipia felis	NCTC12499^{T}	GCA_900445155.1	1035	78.934	451	1986	below_threshold
Afipia carboxidovorans	OM5^{T}; ATCC 49405^{T}	GCA_000021365.1	40137	78.733	438	1986	below_threshold
Afipia carboxidovorans	OM5^{T}	GCA_000218565.1	40137	78.721	438	1986	below_threshold
“Bradyrhizobium acaciae”	10BB^{T}	GCA_020889785.1	2683706	78.528	578	1986	below_threshold
“Bradyrhizobium valentinum”	LmjM3^{T}	GCA_001440405.1	1518501	78.492	571	1986	below_threshold
Bradyrhizobium shewense	ERR11^{T}	GCA_900094605.1	1761772	78.408	569	1986	below_threshold
Bradyrhizobium elkanii	USDA 76^{T}	GCA_023278185.1	29448	78.381	655	1986	below_threshold
“Bradyrhizobium oropedii”	Pear76^{T}	GCA_020889685.1	1571201	78.379	598	1986	below_threshold
Bradyrhizobium viridifuturi	SEMIA 690^{T}	GCA_001238275.1	1654716	78.297	635	1986	below_threshold
Bradyrhizobium betae	CECT 5829^{T}	GCA_024806875.1	244734	78.283	594	1986	below_threshold
Rhodopseudomonas rhenobacensis	DSM 12706^{T}	GCA_014203125.1	87461	78.266	506	1986	below_threshold
Bradyrhizobium retamae	Ro19^{T}	GCA_001440415.1	1300035	78.138	571	1986	below_threshold

Table S4. 16S rRNA gene sequence similarities between *Afipia dichlorophenoxyacetatis* sp. nov. strain DD3^{T} and the type strains of the closely related species

Similarity values based on the 16S rRNA gene sequences were calculated using the pairwise nucleotide sequence alignment tool in EzBioCloud [23] with the DD3^{T} sequence as a query. The data are presented in a descending order of the calculated similarity values.

**Species**	**Strain**	**Accession number ***	**Pairwise similarity (%)**
Bradyrhizobium jicamae	PAC68^{T}	LLXZ01000092	98.23
Bradyrhizobium sediminis	S2-20-1^{T}	MZ315000	98.17
Bradyrhizobium lablabi	CCBAU 23086^{T}	LLYB01000065	98.16
Bradyrhizobium algeriense	RST89^{T}	FJ546419	98.16
Bradyrhizobium erythrophlei	CCBAU 53325^{T}	KF114645	98.13
Bradyrhizobium icense	LMTR 13^{T}	CP016428	98.09
Bradyrhizobium paxllaeri	LMTR 21^{T}	MAXB01000044	98.09
Bradyrhizobium embrapense	SEMIA 6208^{T}	LFIP01000119	98.09
Bradyrhizobium viridifuturi	SEMIA 690^{T}	LGTB01000028	98.09
Bradyrhizobium namibiense	5-10^{T}	KX661401	98.09
Bradyrhizobium mercantei	SEMIA 6399^{T}	MKFI01000007	98.09
Bradyrhizobium murdochi	WSM 1741^{T}	MK676062	98.09
Bradyrhizobium australiense	WSM 1791^{T}	MK676067	98.09
Bradyrhizobium neotropicale	Cp5.3^{T}	AUFA01000009	98.09
Afipia broomeae	ATCC 49717^{T}	KB375282	98.08
Bradyrhizobium cenepequi	CNPSo 4026^{T}	MK676055	98.03
Bradyrhizobium retamae	Ro19^{T}	LLYA01000002	98.02
Bradyrhizobium archetypum	WSM 1744^{T}	MK676065	97.94
Bradyrhizobium septentrionale	1S1^{T}	KP768787	97.86
Bradyrhizobium elkanii	USDA 76^{T}	KB900701	97.80
Bradyrhizobium pachyrhizi	PAC 48^{T}	LFIQ01000091	97.80
Bradyrhizobium tropiciagri	SEMIA 6148^{T}	LFLZ01000084	97.80
Bradyrhizobium ripae	WR4^{T}	MF593081	97.80
Bradyrhizobium ivorense	CI-1B^{T}	CAADFC020000004	97.80
Pseudomonas carboxydohydrogena	DSM 1083^{T}	AB021393	97.72
Afipia clevelandensis	ATCC 49720^{T}	AGWY01000015	97.65
Afipia birgiae	34632^{T}	CAJQ01000091	97.59
Afipia felis	ATCC 53690^{T}	KB375270	97.58
Afipia carboxidovorans	ATCC 49405^{T}	CP002826	97.44
Bradyrhizobium cajani	AMBPC1010^{T}	KY349447	97.30
Bradyrhizobium stylosanthis	BR 446^{T}	KU724142	97.09
Bradyrhizobium shewense	ERR11^{T}	FMAI01000010	97.08
Bradyrhizobium japonicum	USDA 6^{T}	AP012206	97.01
Bradyrhizobium liaoningense	2281^{T}	AF208513	97.01
Bradyrhizobium daqingense	CCBAU 15774^{T}	KJ184551	97.01
Bradyrhizobium americanum	CMVU44^{T}	KU991833	97.01
Bradyrhizobium diversitatis	CNPSo 4019^{T}	MK676046	97.01
Bradyrhizobium subterraneum	58 2-1^{T}	KP308152	96.99
Bradyrhizobium canariense	BTA-1^{T}	AJ558025	96.94
Bradyrhizobium huanghuaihaiense	CCBAU 23303^{T}	HQ231463	96.94
Bradyrhizobium ottawaense	OO99^{T}	NPNY01000075	96.94
Afipia massiliensis	34633^{T}	AY029562	96.64
Bradyrhizobium rifense	CTAW71^{T}	EU561074	96.47
Bradyrhizobium diazoefficiens	USDA 110^{T}	D13430	96.46
Bradyrhizobium betae	NBRC 103048^{T}	AB681928	96.24
Rhodopseudomonas rhenobacensis	Klemme Rb^{T}	AB087719	95.76

* The 16S rRNA gene sequences used here are the same as those used in the phylogenetic analyses (Fig. S1).

Table S5. Putative biosynthetic gene clusters (BGCs) in the DD3^T genome predicted by antiSMASH

Region *	Cluster type †	Size (kb)	Information on most similar known cluster				
			Similarity (%)	Product	Biosynthetic class	MIBiG accession	Size in MIBiG (kb)
1	Terpene	24.7	15	Hopene	Terpene	BGC0000663	13.8
2	Redox-cofactor	22.3					
3	Homoserine lactone	20.6					
4	Terpene	20.9					
5	Linear azol(in)e-containing peptide	23.8					

* Regions identified by antiSMASH version 7.0.0 [40] using the "relaxed" setting.

† Abbreviations shown here are explained in the antiSMASH glossary available at https://docs.antismash.secondarymetabolites.org/glossary/#terpene.

Table S6. Types and numbers of putative biosynthetic gene clusters (BGCs) in the genomes of DD3^T and its closest relatives predicted by antiSMASH

Strains: 1, *Afipia dichlorophenoxyacetatis* DD3^T; 2, *Afipia broomeae* ATCC 49717^T; 3, *Afipia massiliensis* DSM 17498^T; 4, *Afipia birgiae* 34632^T; 5, *Afipia clevelandensis* ATCC 49720^T; 6, *Afipia felis* ATCC 53690^T; 7, *Afipia carboxidovorans* OM5^T.

**Cluster type** *	**Number of putative biosynthetic gene clusters †**						
	**1**	**2**	**3**	**4**	**5**	**6**	**7**
Acyl amino acids, homoserine lactone		1					
Homoserine lactone	1	2	1			1	3
Linear azol(in)e-containing peptide	1						
Non-ribosomal peptide synthetase						2	
Redox-cofactor	1	1	1	1	1		
RiPP-like		1			1	1	
RiPP-recognition-element-containing cluster					1		
RiPP-recognition-element-containing cluster, lassopeptide							1
Terpene	2	2	2	2	2	2	2

* Abbreviations shown here are explained in the antiSMASH glossary available at https://docs.antismash.secondarymetabolites.org/glossary/#terpene.

† Putative BGCs identified by antiSMASH version 7.0.0 [40] using the “relaxed” setting.
